# New Derivatives of Modified Starch for Food Technology

**DOI:** 10.3390/molecules29143292

**Published:** 2024-07-12

**Authors:** Emilia Konował, Joanna Sulej-Chojnacka, Krystyna Prochaska

**Affiliations:** 1 Institute of Chemistry and Technical Electrochemistry, Poznan University of Technology, Berdychowo 4, 60-965 Poznan, Poland; emilia.konowal@put.poznan.pl; 2 Materials Engineering Division, Łukasiewicz Research Network–Poznan Institute of Technology, Ewarysta Estkowskiego 6, 61-755 Poznan, Poland; joanna.sulej-chojnacka@pit.lukasiewicz.gov.pl; 3 Institute of Chemical Technology and Engineering, Poznan University of Technology, Berdychowo 4, 60-965 Poznan, Poland

**Keywords:** chemically modified starch, enzymatic hydrolysis, recirculated membrane reactor, rheological properties, water-binding capacity

## Abstract

The food industry extensively uses chemically modified starches and their hydrolysates, which is mainly due to their emulsification ability. Therefore, it becomes inevitable to develop new starch derivatives, including modified starch hydrolysates, and effective preparation methods to meet the increasing demands of producers, consumers, and technology. This study comprehensively researches the physical, chemical, and functional properties (such as the water-binding capacity, swelling power, solubility, and fat absorption capacity) of chemically modified biopolymers and their enzymatic hydrolysis products. We utilized oxidized and acetylated potato and waxy-corn starches with varying degrees of substitution by carboxyl and acetyl groups in our research. The process of enzymatic hydrolysis was performed in a recirculated membrane reactor (CRMR). Our findings indicated that the physicochemical properties of starch derivatives and their hydrolysates depended on the biological origin of the biopolymer and the type and degree of modification. However, the presence of carboxyl groups in the modified starch molecules is critical and affects the rheological properties and water-binding capacity of the starch preparations. For example, in the case of waxy-corn starch preparations with a lower content of carboxyl groups (i.e., derivatives with a low degree of oxidation), the water-binding capacity (WBC) increases when compared to native starch. The highest WBC value of 206.3% was noted for the doubly modified waxy-corn starch with an oxidation degree of 0.2% and an acetylation degree of 2.5%, while native waxy-corn starch shows a WBC of 161.4%. In contrast, it was observed that preparations with a higher content of carboxyl groups, i.e., derivatives with an oxidation degree of 2.5%, show a lower swelling power compared to native waxy starch.

## 1. Introduction

Starch is a highly valuable natural resource due to its widespread availability, low cost of acquisition, biodegradability, biocompatibility, and ease of modification. Its economic significance is further enhanced through processing, which allows for the production of a wide range of derivatives with varied chemical, physicochemical, and functional properties. Modifying starch can involve enzymatic, chemical, and physical methods. It is important to note that the specific modification methods affect properties such as the fluidity, durability, temperature resistance, and susceptibility to further processing [1,2,3,4,5,6].

Starch hydrolysis can occur through the use of acids (acid hydrolysis) or enzymes (enzymatic hydrolysis) [7]. Enzymatic starch hydrolysis takes place under gentle conditions, enabling the creation of products with precise physical and chemical properties. This process is distinguished by its specificity of action and is a highly efficient alternative to acid hydrolysis.

Enzymes used in food technology typically have a degradative, or catabolic, nature, usually involving the hydrolytic breakdown of substrates. α-Amylase (4-α-glucanohydrolase α-1,4-glucan) belongs to the group of endoamylases. This enzyme randomly attacks α-1,4-glycosidic bonds within the starch molecule, resulting in a rapid decrease in the viscosity of the starch paste. During the progression of enzymatic hydrolysis, large-sized dextrins are initially formed, which then break down into smaller-sized dextrins until the reaction finally ends with the formation of limit dextrins [8].

Enzymatic starch hydrolysis typically occurs in a traditional batch stirred-tank reactor, with two main stages: liquefaction and saccharification. However, this method has several drawbacks, including low efficiency, the need for different enzymes in each stage, the requirement for enzyme separation and deactivation after each use (which significantly increases production costs), single use of enzymes, extended reaction times (24–48 h), and large reactor volumes. While using enzymes from the α-amylase group, such as Termamyl, allows for the enzymatic hydrolysis of native starch to dextrins in a single step in the stirred-tank reactor, the resulting product requires further expensive purification. To address these challenges, one proposed approach is the utilization of a continuous recycled membrane reactor (CRMR) to reduce the environmental impact of chemical processes [9,10].

In starch processing, membrane reactors with a biocatalyst in solution are commonly used, specifically reactors with an external separation unit. In such reactors, enzymes or microorganism cells are present in the reagent solution in their native state, thus retaining their natural catalytic activity. When their sizes are too small relative to the pore sizes of the membrane, they are immobilized on special carriers or form larger aggregates through crosslinking. An advantage of these reactors is the easy replacement of deactivated catalysts. During membrane separation processes (microfiltration and ultrafiltration), a decrease in permeate flux over time is observed, which is mainly caused by concentration polarization, fouling, or the adsorption of separated components on the membrane surface. Moreover, high shear stresses and rapid circulation of the reaction mixture in the system can additionally accelerate enzyme deactivation [9,11,12,13,14,15].

Several potential applications of recirculating membrane reactors in starch processing have been described in the literature. For instance, Słomińska et al. [11] pointed out the possibility of using an ultrafiltration membrane reactor in the continuous production of cyclodextrins. The research material consisted of soluble potato starch. The studies showed greater efficiency of the process conducted using a membrane reactor compared to a stirred-tank reactor and allowed for the optimization of the cyclodextrin production process in a recirculating membrane reactor. D. Paolucci-Jeanjean et al. [12] investigated the effect of operating parameters of enzymatic starch hydrolysis in a continuous recycled membrane reactor (CRMR) on changes in enzyme activity throughout the process. The research material consisted of native cassava starch, and the enzymatic preparation used in the experiments was Termamyl 120L. The authors demonstrated that the main factors causing the fouling of ultrafiltration membranes and enzyme deactivation are adsorption on the membrane surface and in the membrane pores.

Sarbatly et al. [13] outlined the potential of a CRMR reactor for producing pure and sterile glucose solution on site. They used native tapioca starch as the raw material and employed two types of enzymes from the amylase and amyloglucosidase groups. The results indicated that the membrane process (while maintaining appropriate process parameters) was three times more efficient than the process conducted in a stirred-tank reactor, Grześkowiak and Słomińska [14] conducted an enzymatic hydrolysis of maltodextrin using a continuous recycled membrane reactor with ceramic tubular modules (cut-off 5 and 8 kDa) and hollow-fiber modules (cut-off 6 kDa). The authors demonstrated greater efficiency in maltose production using ceramic membranes while noting that the fouling of organic membranes was three to four times lower than that of ceramic membranes.

Słomińska et al. [15] conducted a study on optimizing the enzymatic hydrolysis process of maltodextrin in a membrane reactor with an external ultrafiltration separation unit equipped with a ceramic membrane with different separation capabilities. The highest product concentration and the smallest reduction in permeate flux during the process were observed for the membrane with a cut-off of 5 kDa. Additionally, the steady state of the membrane reactor during the hydrolysis process was achieved 2–3 h after the start of the reaction.

Paolucci-Jeanjean et al. [9] demonstrated that using a CRMR with a membrane cut-off of 50 kDa for starch hydrolysis retains the enzyme and unhydrolyzed derivatives in the reaction environment while allowing the hydrolysis products to leave the system freely. This is an extremely important property from the perspective of starch hydrolysis because, as shown by the authors of the cited work, the presence of glucose and maltose in the reaction mixture has an inhibitory effect on amylolytic enzymes.

Unfortunately, there is a limited amount of research available on the enzymatic hydrolysis of modified starches in membrane reactors. Previous studies have focused on the potential use of recirculating membrane reactors in the enzymatic hydrolysis of oxidized starch [16,17] and sodium octenylsuccinate starch [18,19,20]. These works sought to optimize the parameters of enzymatic hydrolysis of modified starches in CRMR and assess the impact of these parameters on the functional properties of the resulting hydrolysates.

In this study, we present the preparation and evaluation of the functional properties of starch preparations modified by oxidation and acetylation as well as their hydrolysates obtained in CRMR.

## 2. Results and Discussion

### 2.1. Analysis of the Degree of Acetyl Group Substitution

**Figure 1:**
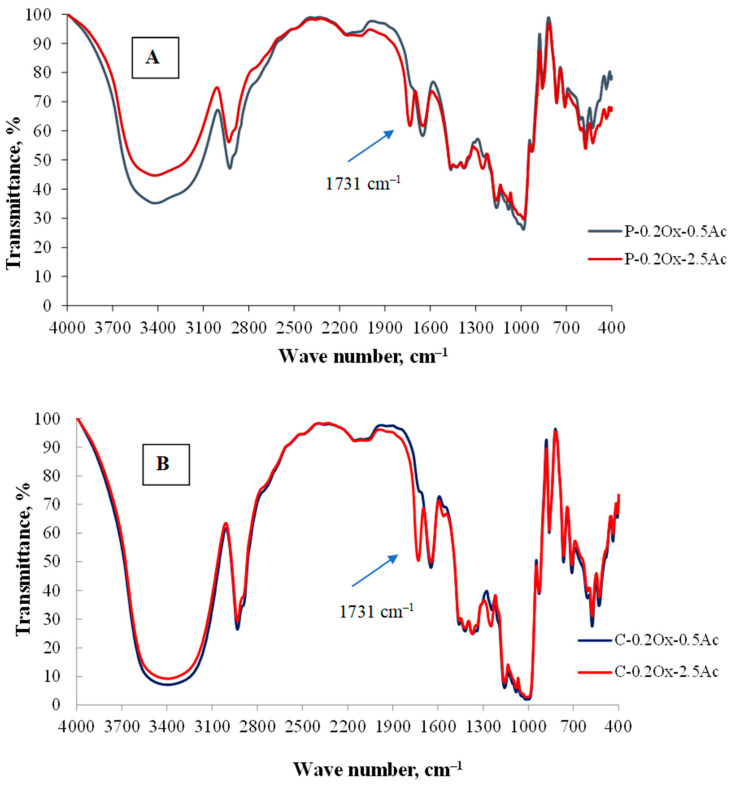
FT-IR spectra of acetylated starch and oxidized starch derived from (**A**) potato, (**B**) waxy-corn.

To confirm the presence of acetyl groups in the obtained derivatives, the macromolecular structure of the starch was analyzed using FT-IR spectral analysis. Figure 1 shows the FT-IR spectra of starch derivatives with a low degree of oxidation (0.2 Ox) and varying acetyl group content for potato starch (Figure 1A) and waxy-corn starch (Figure 1B).

The band appearing at a wavelength of 1731 cm^−1^ in the obtained spectra is attributed to the stretching vibrations of the carbonyl (C=O) group of esters, indicating the formation of ester carbonyl groups in the newly synthesized starch products during the esterification reaction. The intensity, area, and height of the peaks are strongly dependent on the degree of substitution (DS) of the derivatives studied. As the DS increases, the intensity, area, and height of the peaks around the bands at approximately 3395 cm^−1^, 1081 cm^−1^, and 1014 cm^−1^, particularly those associated with O–H stretching vibrations, gradually weaken. This indicates that hydroxyl groups fully participate in the substitution reaction. With the increase in DS, there is an observed increase in the intensity, area, and height of the peaks corresponding to the presence of methyl groups in the acetyl group.

The absence of peaks in the 1850–1760 cm^−1^ range confirms that the final product was free from unreacted acetic anhydride. Additionally, no absorption band for the carboxyl group was observed in the 1700 cm^−1^ range, confirming that the product was free from the by-product of the reaction, acetic acid. The obtained FT-IR absorption spectra of acetylated starches are consistent with the spectra described in the literature [21,22].

To quantitatively determine the degree of substitution of starch preparations with acetyl groups, alkacimetric titrations were performed according to FAO/WHO Smith’s method [23]. The results of the determination of the degree of substitution with acetyl groups for the double-modified starch preparations using Smith’s method are summarized in Table 1.

The data analysis shows differences in the acetyl group content among the various derivatives. The biopolymers were found to have acetyl group substitutions of 0.5% and 2.5%, respectively. Therefore, terms such as biopolymers with high or low degrees of substitution, or those with small or large acetyl group content, are suitable for use in the subsequent parts of the study.

### 2.2. Microscopic Analysis of Starch Preparations

**Figure 2:**
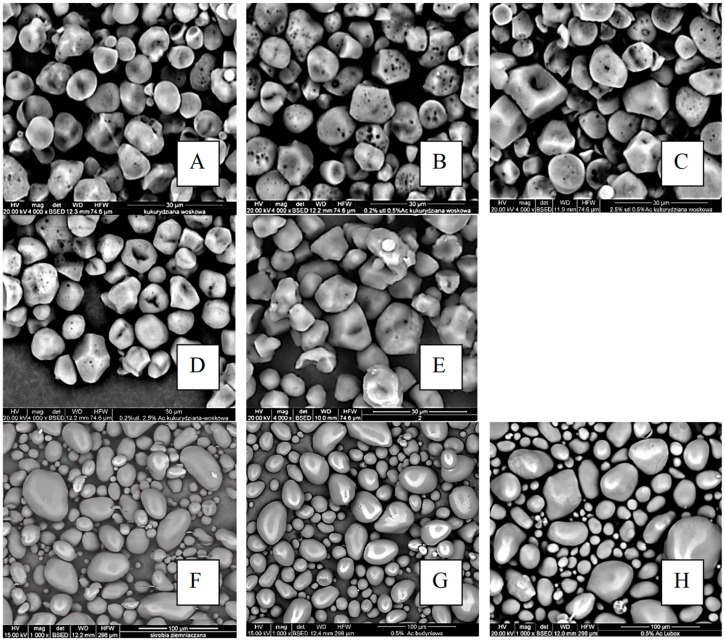
Micrographs of starch granules (magnitude ×4000): (**A**) native waxy-corn starch, (**B**) C-0.2Ox-0.5Ac, (**C**) C-2.5Ox-0.5Ac, (**D**) C-0.2Ox-2.5Ac, (**E**) C-2.5Ox-2.5Ac and micrographs of starch granules (magnitude ×1000): (**F**) native potato starch, (**G**) P-0.2Ox-0.5Ac, (**H**) P-2.5Ox-0.5Ac.

Micrographs of starch granules are presented in Figure 2. The granules of waxy-corn starch (Figure 2A–E) exhibit pentagonal and hexagonal shapes with irregular surfaces. The majority of the granules exhibit uneven depressions and pores on their surfaces; however, some granules have a completely smooth surface. Microscopic analysis of potato starch granules (Figure 2F–H) confirmed their oval shape and smooth surface. According to the literature data [24,25], the presence of pores on the surface of starch granules may affect their susceptibility to enzymatic hydrolysis, as these pores serve as points of enzymatic attack.

Waxy-corn starch preparations obtained through chemical modification exhibit changes primarily in the central part of the grain, which is the growth point and less organized, making it more susceptible to modification agents (Figure 2B–E). The structure of starch granules did not deform due to the action of sodium chlorate(1) and acetic anhydride [26]. In the case of potato starch (Figure 2F–H), modification with acetic anhydride and sodium chlorate(1) did not cause visible changes in the morphology of the granules.

It has been postulated that the morphology of chemically modified granules is dependent upon the botanical source of the starch. The susceptibility of starch to modification is attributed to the physiology of starch granules. As reported by Singh et al. [27], the morphology of hydroxypropylated potato starch undergoes a transformation in the central area (core) of the granule. Nevertheless, this transformation was not observed in corn starch. Similarly, Lawal and Adebowale [28] did not observe significant differences in the shape and appearance of native and modified acetylated bean starch. No structural changes were observed for chemically modified waxy-potato starch, either. Additionally, the authors of the paper [29] demonstrated that crosslinking with acetylated adipinate does not result in any adverse effects on the morphology of the granules.

In the case of starch preparations obtained through oxidation and acetylation reactions, there is no observed tendency for starch granules of both tested starches to aggregate (stick together). Different observations were made by Lin et al. [30,31]. According to the cited authors, modifications lead to changes in the morphology of the granules. Additionally, the surface of the granules becomes rougher. Similarly, Xie and colleagues [32] observed changes in the morphology of starch granules modified with citric acid. The authors demonstrated that chemical modification with citric acid can lead to the loosening of organized areas in the central part of the starch granule by creating channels and depressions.

### 2.3. Viscosity of Starch Paste

It was found that the chemical modification of waxy-corn and potato starch through simultaneous oxidation and acetylation results in significant changes in the rheological properties of suspensions/solutions formed by the starch preparations. The values of dynamic viscosity of 5% starch pastes, measured at a shear rate of 300 rpm for both potato and waxy-corn starch, as presented in Table 2, clearly indicate that with an increasing degree of substitution of the starch macromolecule with carboxyl groups, the viscosity of the solutions decreases. It has been demonstrated that starches that have undergone oxidation and acetylation, with an oxidation degree of DS equal to 0.04 (i.e., P-0.2Ox-0.5Ac and P-0.2Ox-2.5Ac), exhibit high viscosity, approximately 0.2 Pa·s, whereas for waxy-corn starch preparations, these values are almost twice as high (with a viscosity of 0.41 Pa·s for C-0.2Ox-0.5Ac and 0.33 Pa·s for C-0.2Ox-2.5Ac). The starch preparation with a high carboxyl group content, P-2.5Ox-2.5Ac, has a dynamic viscosity three orders of magnitude lower than the derivatives with a low degree of oxidation. 

Studies on the rheological properties of doubly modified starch pastes showed that the viscosity of the paste is significantly influenced only by the degree of oxidation of the starch preparation. The introduction of acetyl substituents into the starch macromolecule causes only a slight change in the solution’s rheology for both potato and waxy-corn starch.

The viscosity of native starch pastes strongly influences the hydrodynamic parameters of membrane reactor operation. In addition to biofouling, it is the most important factor in the variability of hydrolysis yields. Understanding the rheological properties of starch and its derivatives is crucial because it provides insights into the behavior of polysaccharides in various processes, particularly in the food industry. An understanding of the behavior of starch gels is of paramount importance for the regulation of production processes and the optimization of the stability and sensory characteristics of end products. The rheological properties of starch solutions also influence the quality of end products in the paper and textile industries.

**Figure 3:**
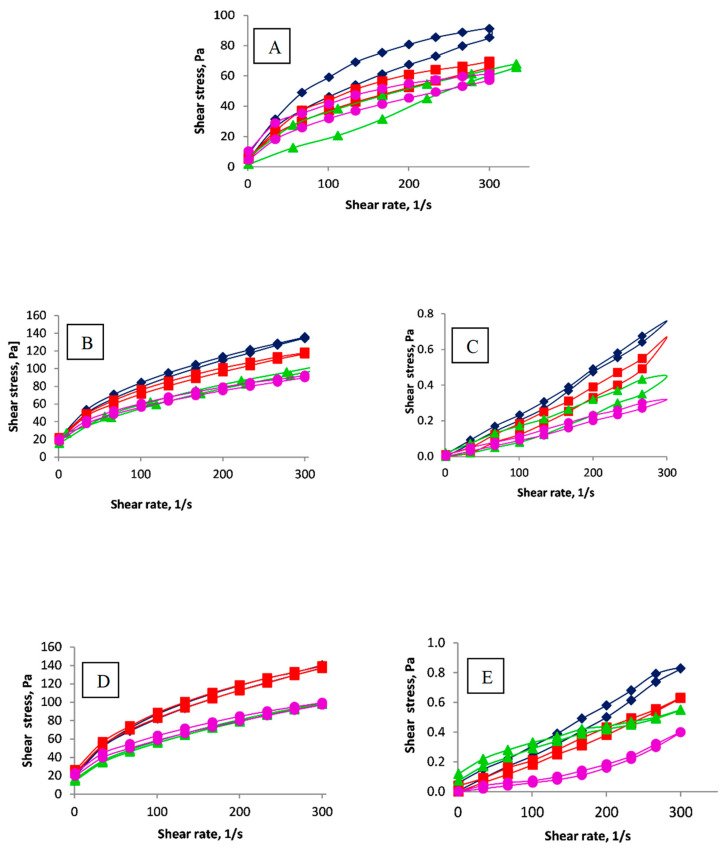
Flow curves of (**A**) native waxy-corn starch, (**B**) C-0.2Ox-0.5Ac, (**C**) C-2.5Ox-0.5Ac, (**D**) C-0.2Ox-2.5Ac, (**E**) C-2.5Ox-2.5Ac at temperature: ♦ 30, ■ 40, ▲ 60 and ● 75 °C.

Knowledge of the rheological properties of starch paste is essential for effectively creating certain products. Temperature during distribution and storage before consumption is a critical environmental factor that can affect the final stability and required textural characteristics of starch dispersions. Therefore, establishing the influence of temperature on the rheological properties of starch dispersions helps predict and control changes in the physical properties of starch-derived products. The effect of temperature on viscosity can be illustrated, for example, by modifications of waxy-corn starch (Figure 3).

Lower viscosity values for starch derivatives are observed at higher temperatures. It is widely accepted that the viscosity of a liquid is influenced by intermolecular forces that restrict molecular movement. At higher temperatures, there is an increase in the kinetic energy of dispersion. Consequently, polysaccharide molecules become more flexible, aiding in the “unravelling” of their long chains, which reduces the system’s viscosity [33].

The analysis of the flow curves of starch derivatives with a low degree of oxidation (Figure 3B,D) reveals that the character of the flow curves remains consistent at different temperatures. The graphs in Figure 3 demonstrate that for all derivatives, regardless of the carboxyl group content, the modification process did not alter the nature of the flow curves in comparison to native waxy-corn starch.

### 2.4. Enzymatic Hydrolysis of Modified Starch

Analysis of the viscosity changes in the retentate stream during the enzymatic hydrolysis of doubly modified potato starch P-0.2Ox-0.5Ac (Table 3) indicates that the viscosity of the starch paste drastically decreased (nearly 50-fold) during the initial 60 min of the process and then stabilized. Similar observations were made for waxy-corn starch.

It can be concluded that the viscosity of the starch solution was responsible for process efficiency only in the initial stage of hydrolysis in the CRMR. The observed decline in filtrate flux during the later stages of hydrolysis is likely attributable to the progressive biofouling of the ultrafiltration membranes.

**Figure 4:**
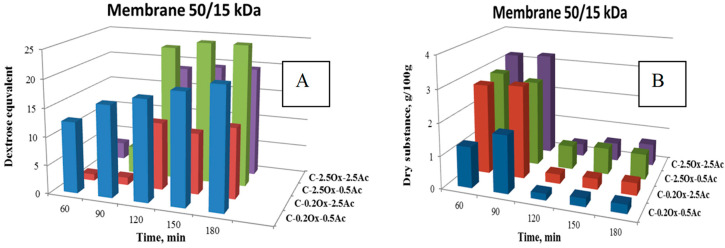
Change in the value of glucose equivalent (**A**) and dry substance content (**B**) over time for the hydrolysis products of waxy-corn starch derivatives.

Figure 4 presents the DE values and changes in dry matter content in the filtrate fraction determined during the enzymatic hydrolysis of waxy-corn starch derivatives in a CRMR reactor, which was equipped with two external ultrafiltration modules and membranes with 50 and 15 kDa cut-off values.

Upon analysis of the presented data, it was observed that the efficiency of the hydrolysis process is influenced by the content of both acetyl and carboxyl groups in the hydrolyzed starch molecule. The hydrolysates obtained from derivatives with a higher content of acetyl groups exhibited a lower degree of saccharification compared to those with a lower content of acetyl groups. Specifically, the degree of saccharification values determined after 180 min of hydrolysis differed by approximately 10 DE units. The C-2.5Ox-0.5Ac derivative exhibited the highest efficiency in the hydrolysis process.

Additionally, it was observed that the C-0.2Ox-0.5Ac derivative is particularly susceptible to the action of the amylolytic enzyme. After 90 min of exposure to α-amylase, this derivative achieved a degree of saccharification of 12.58 DE, indicating that its susceptibility to hydrolysis is more than four times higher compared to the other three double-modified derivatives.

The dry matter values of the filtrates shown in Figure 4B indicate that during the first 90 min of the enzymatic hydrolysis process, the C-2.5Ox-2.5Ac double-modified preparation exhibited slightly higher dry matter content compared to the other derivatives. However, during the second separation step using a 15 kDa cut-off membrane, a significant reduction in the dry matter content of the filtrate fraction was observed for all considered systems. This phenomenon can be explained by the fact that during the initial stage of separation with the membrane of cut-off of 50 kDa, products with higher molecular weight were separated.

Furthermore, it was observed that in the case of waxy-corn starch, as the content of acetyl groups in the hydrolyzed derivative molecule increases, the degree of saccharification of the obtained hydrolysate decreases. Conversely, an increase in the content of carboxyl groups in the hydrolyzed biopolymer molecule, specifically at 2.5% -COOH content, increased the efficiency of the enzymatic hydrolysis process.

**Figure 5:**
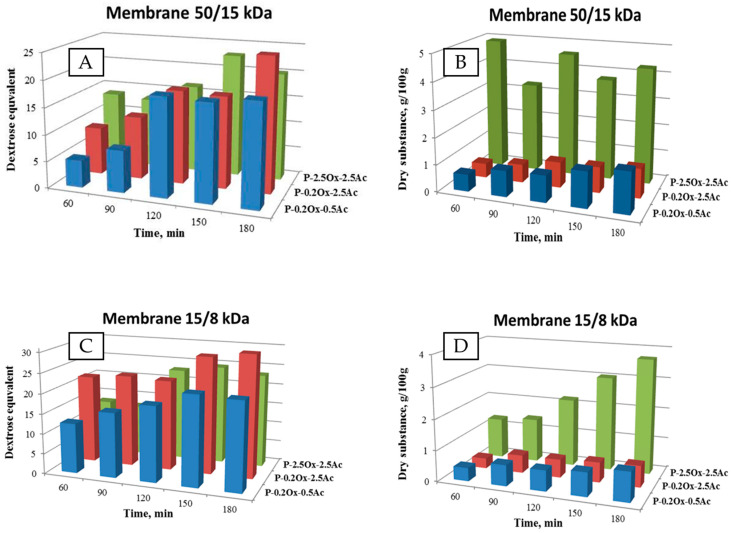
Change in the value of glucose equivalent (**A**,**C**) and dry matter content (**B**,**D**) over time for the hydrolysis products of potato starch derivatives.

The comparative analysis of the saccharification degree of the hydrolysates of potato starch tested (Figure 5A,C) indicates that, as expected, the CRMR membrane reactor equipped with two UF modules with 15 and 8 kDa cut-off membranes produces products with a higher degree of saccharification compared to the 50 and 15 kDa membrane configuration. The 50/15 kDa configuration allows for higher volume flows of filtrates, resulting in a greater volume of the final dry product.

The highest amounts of dry matter (Figure 5B,D) were obtained in the case of the P-2.5Ox-2.5Ac derivative hydrolysis, which is related to the higher initial concentration of this derivative in the reactor feed. It is also worth noting that in the case of a derivative with a lower content of acetyl groups, the content of the dry substance in the permeate is higher than in the case of hydrolysis of a more highly acetylated derivative (P-0.2Ox-2.5Ac).

Therefore, the most efficient and effective hydrolysis process for starch that has been doubly modified by oxidation and acetylation is the two-stage filtration on 50 and 15 kDa cut-off membranes in the CRMR.

### 2.5. Physicochemical Properties of Starch Hydrolysates

A series of tests were conducted on the starch preparations and their hydrolysates in order to ascertain their properties and potential applications. The tests included an analysis of the oil absorption capacity, water solubility, swelling strength in water, and foam-forming properties.

#### 2.5.1. Fat Absorption Capacity

Hydrolysates of modified waxy-corn and potato starch derivatives obtained by enzymatic hydrolysis were subjected to fat adsorption studies. The fat absorption capacity (FAC) was assessed from the perspective of the potential use of the obtained hydrolysates as functional components in food products.

**Figure 6:**
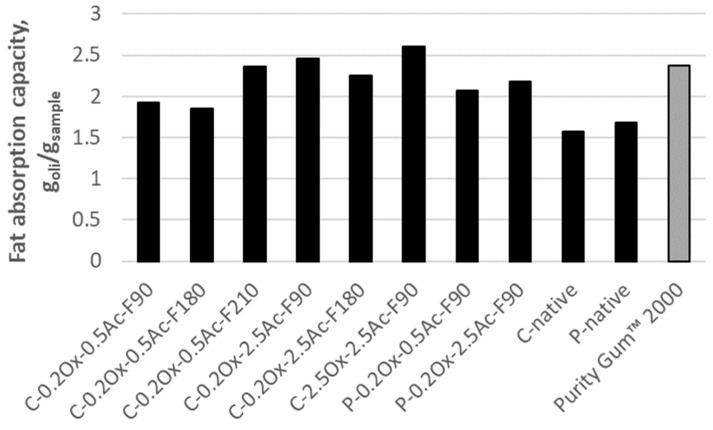
The fat absorption capacity of starch hydrolysates of potato starch and waxy-corn starch.

Analysis of the results obtained for potato starch hydrolysates doubly modified by oxidation and acetylation, as shown in Figure 6, allows us to state that the addition of more acetyl substituents increases the hydrophobicity of the preparations. The average FAC value for the hydrolysate with a degree of substitution with ester groups equal to 0.5% was 2.08 g oil/g sample for the potato starch, while for the filtrate with a 2.5% content of carboxyl groups, this value increased to 2.16 g oil/g sample.

The obtained FAC results for potato starch and its hydrolysates indicate that the hydrolysates have a higher fat absorption capacity than the native starch. The improvement in fat absorption capacity, and thus the increased lipophilicity of the modified derivative compared to native starch, is a direct result of the introduction of functional groups into the starch molecule.

Figure 6 also shows the FAC values for selected hydrolysates of modified waxy-corn starch derivatives obtained by enzymatic hydrolysis in a CRMR reactor at different process times. 

The oil absorption potential of the hydrolysates of the double-modified derivatives varied from 1.85 to 2.60 g oil/g sample with the highest fat absorption capacity demonstrated by the C-2.5Ox-2.5Ac hydrolysate of waxy-corn starch (2.60 g oil/g sample). The obtained results indicate that hydrolysates with a higher degree of substitution with acetyl groups have a higher FAC than hydrolysates with a lower degree of substitution. The improvement in fat absorption capacity is a result of the introduction of functional groups into the starch macromolecule, which facilitates the binding of oil molecules. Additionally, it was observed that the duration of the enzymatic hydrolysis process affects the fat absorption capacity of the obtained hydrolysates.

Comparing the oil absorption values for commercial Purity Gum™ 2000 (FAC = 2.37 g oil/g sample) and the FAC values for the waxy-corn and potato starch hydrolysates obtained in this study, it was found that only the hydrolysates of the C-0.2Ox-2.5Ac and C-2.5Ox-2.5Ac derivatives, obtained after 90 min of hydrolysis (F90), have a higher FAC value than the commercial preparation. 

Adebowale et al. [18] analyzed the effect of oxidation and acetylation of starch from Canavalia gladiata on the FAC value, and they showed that as a result of the modification, the fat absorption capacity of the obtained preparations increased. Starch treated with oxidation showed the highest fat absorption capacity with the FAC value for oxidized starch rising from 2.9 to 3.6 g/g relative to native starch. Similar conclusions were presented by Lawal [34], who also showed that the biopolymer modification process increases the fat absorption capacity.

In the paper [35], the authors showed that for starch obtained from mungo beans, the introduction of carboxyl and acetyl groups resulted in a nearly 2.5-fold increase in the FAT index. However, literature reports on the effect of the type of polysaccharide modification on FAC values are not conclusive. For example, Sathe and Salunkhe [36], when studying the modified bean starch, showed that incorporating carboxyl and acetyl groups into the starch molecule did not improve the fat absorption capacity of the tested starch preparations.

#### 2.5.2. Water-Binding Capacity

**Figure 7:**
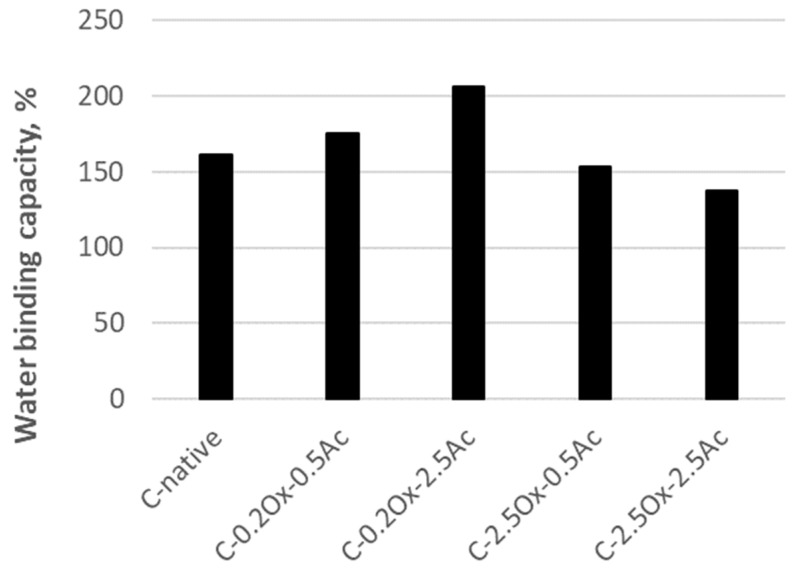
The binding capacity of water of waxy-corn starch and modified starches.

The water-binding capacity (WBC) refers to the amount of water that a starch molecule can hold. Figure 7 illustrates the water-binding capacity for native waxy-corn starch and modified starches.

Native waxy-corn starch has a water-binding capacity of 161.4%. Starch formulations with a lower content of carboxyl groups demonstrate an increased water retention capacity compared to the original starch. The highest water-binding capacity is observed in double-modified waxy-corn starch with a degree of oxidation of 0.2% and a degree of acetylation of 2.5%, achieving a WBC value of 206.3%. On the other hand, preparations with a higher content of carboxyl groups, such as C-2.5Ox-0.5Ac and C-2.5Ox-2.5Ac derivatives, show a lower water-binding capacity in comparison to the initial waxy-corn starch.

Similar relationships for acetylated corn starch were observed by Garg and Jana [37]. They stated, that for starch with a higher content of carboxyl groups (i.e., 2.5%) and its derivatives additionally subjected to an acetylation reaction, the water retention capacity is lower than that of the original native waxy-corn starch. Additionally, Diop, Li, Xie, and Shi [38] showed that as the degree of substitution increases, the water absorption capacity of acetylated corn starch declines. Ozturk, Koksel, and Ng [39] investigated the water-binding capacity of corn starch with 55% and 70% amylose content and their derivatives obtained by acid hydrolysis. The modified starches were found to have almost twice the water-binding capacity compared to the native starch. From the presented WBC values for modified derivatives of waxy-corn starch, it can be concluded that the content of carboxyl groups is the decisive factor for the starch’s ability to bind water.

#### 2.5.3. Gelatinization

Gelling is an important property of starch biopolymers. To gain insight into the structural and molecular differences between starch granules, the solubility and swelling capacity of starch were analyzed. The swelling of starch granules is associated with the gelatinization of starch during heating over water. The swelling force (SF) is defined as the mass ratio of the resulting gruel to the dry mass of the initial sample. According to Li and Yeh [40], the swelling of starch granules is a property of amylopectin. The onset of swelling and gelatinization is determined by the crystallites of the branched glucose polymer.

**Figure 8:**
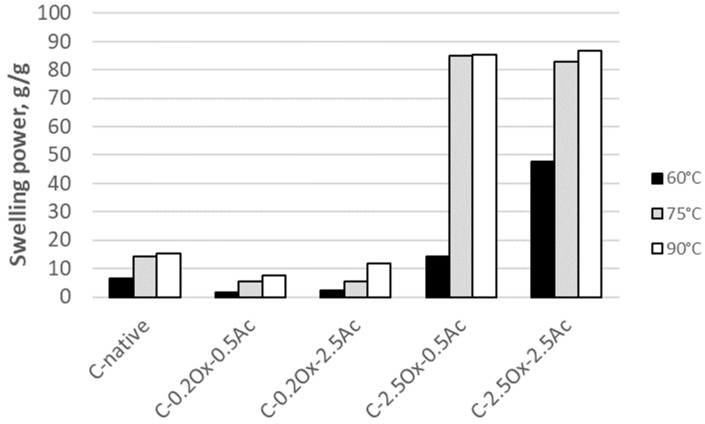
The swelling force of native waxy-corn starch and starch modified at various temperatures.

The swelling force of native and chemically modified waxy-corn starch is shown in Figure 8. It has been observed that the swelling force increases with temperature. The greatest increase in SF value is observed at 75 °C and 90 °C for highly oxidized starch and its acetylated derivatives. As the temperature rises from 60 to 75 °C, the swelling force of the test preparations exhibits a significant increase, from 14.1 to 84.9 g/g for C-2.5Ox-0.5Ac starch and from 47.7 to 86.8 g/g for C-2.5Ox-2.5Ac starch. However, both the temperature increase from 75 to 90 °C and the acetylation process result in only slight alterations to the swelling force.

According to Luo et al. [29], modified starches, such as hydroxypropylated ones, demonstrate an augmented swelling force. This increase is related to the reduction in interactions between glucan chains due to the rise in hydrophilicity of starch upon the introduction of hydrophilic groups. Furthermore, the intermolecular and intramolecular hydrogen bonds of the glucan are disrupted, thereby weakening the structure of the starch granules. Singh, Chawla, and Singh [41] elucidated the phenomenon of increased swelling force due to the presence of hydrophilic groups that retain water. Additionally, the authors demonstrated that native potato starch has a greater swelling force (20.3%) compared to native corn starch (10.3%). The authors attributed this discrepancy to the differing sizes of the granules and the weak internal organization. The low value of the swelling force for corn starch is attributed to the presence of lipids.

The impact of crosslinking time on the swelling capacity of modified waxy starch was presented by Kurakake et al. [42]. The researchers found that as the time and degree of crosslinking increased, the swelling capacity of the starch granules decreased from 39.8 to 8.8 g/g after 10 h of reaction.

#### 2.5.4. Solubility

**Figure 9:**
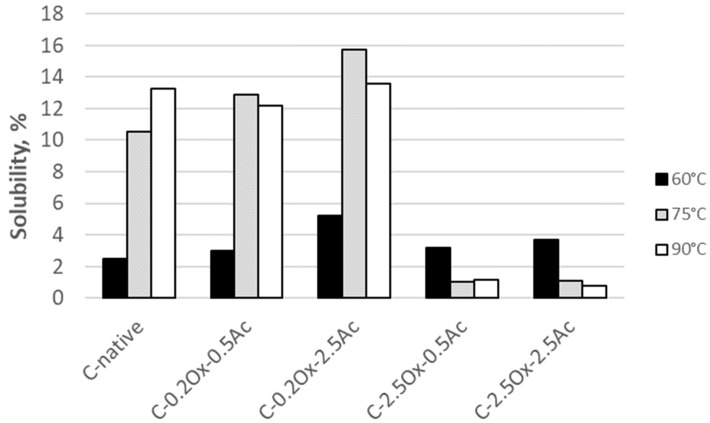
The solubility of native waxy-corn starch and starch modified at various temperatures.

Solubility (S) is defined as the ratio of the amount of dissolved starch in the supernatant compared to the dry weight of the original sample. Figure 9 illustrates the solubility of native waxy-corn starch and its oxidized and oxidized-acetylated derivatives. The solubility of native waxy-corn starch increases with rising temperature, following the results for acetylated corn starch preparations previously described in the literature [41]. However, other researchers have reported an increase in solubility due to the acetylation reaction in their studies as evidenced by the findings presented in [41,43]. This is in contrast to our findings, which show the highest solubility (15.7%) at 75 °C for the C-0.2Ox-2.5Ac derivative. Additionally, our results indicate that starches with a low degree of substitution with carboxyl groups exhibit greater dissolution capacity compared to native waxy-corn starch, which is a trend also observed by Garg and Jana [37] in their study on acetylated corn starch.

As shown in Figure 9, the studied derivatives exhibited the greatest solubility at 75 °C. An increase in temperature by 15 °C results in a reduction in the solubility of the aforementioned starch by approximately 2%. Polysaccharide formulations with a higher content of carboxyl groups (i.e., 2.5% Ox at 60 °C) exhibited solubility characteristics comparable to those of the starting starch. It can be observed that an increase in temperature leads to a decrease in the solubility of these preparations.

Native starch does not dissolve in water at ambient temperature due to its structure. Starch granules start to integrate with water only at high temperatures. The amount of acetyl groups in starches with a low degree of substitution (DS) is insufficient to change the behavior of hydroxyl groups. In starch preparations with a high degree of substitution, the acetyl groups replace the majority of the hydroxyl groups in the starch molecule, thereby reducing its interaction with water. Consequently, at high DS, the solubility and water-binding capacity decrease compared to native waxy-corn starch. These results show that the solubility and water-binding ability depend on two effects: (1) opening the structure of the low-acetylated starch macromolecule, making it more accessible to water, and (2) increasing the hydrophobic nature of polymer chains, which increases gradually as the degree of substitution rises [37].

#### 2.5.5. Foam Stability

In the next stage of the research, for selected starch hydrolysates, an attempt was made to determine the foaming ability of the hydrolysis products and to measure the volume and stability of the foam according to the method by Lawhon, Carter, and Matil [44]. However, it was found that the analyzed amylolytic hydrolysis products did not exhibit any foaming properties. The foam generated during vigorous mixing collapsed within a few seconds, making it impossible to measure both the volume and stability of the foam.

## 3. Experiment

### 3.1. Materials

PPPZ Poland graciously provided native and oxidized potato and waxy-corn starch. Additionally, POCh (Gliwice, Poland) supplied hydrochloric acid, sodium hydroxide, nitric acid, copper(II) sulfate pentahydrate, potassium sodium tartrate, sulfuric acid, sodium thiosulfate concentrate, potassium iodide, and soluble starch, all of which were of analytical grade and used without purification. Finally, Novozymes (Bagsvaerd, Denmark) kindly provided BAN 480L, which is an α-amylase produced by selected strains of *Bacillus amyloliquefaciens*.

### 3.2. Methods

Fourier-transform infrared (FT-IR) analyses were performed using a Bruker FT-IR IFS 113v spectrometer (Bruker Optics, Billerica, MA, USA) in the mid-infrared range of 4000 to 400 cm^−1^ (resolution 2 cm^−1^). The viscosity of starch solutions was measured using a Haake Viscotester VT 550 (Thermo Scientific™, Waltham, MA, USA) viscometer equipped with an MV-DIN rotor at a temperature of 60 °C.

The native waxy-corn and potato starch, as well as their chemically modified derivatives with varying levels of carboxyl and acetyl groups, were subjected to microscopic examination. The structure of native starch granules and chemically modified starches was observed using a scanning electron SEM microscope S-3400N, 20,000 kV (Hitachi, Tokyo, Japan).

The determination of the degree of substitution with acetyl groups was carried out using the method according to Smith [45,46].

The fat absorption capacity (FAC) was measured as follows: 0.5 g of starch was placed in a 50 mL centrifuge tube, and 10 mL of refined sunflower oil was added. The contents of the tube were vigorously mixed for 5 min using a mechanical stirrer and then centrifuged at 4500× *g* for 30 min. The unbound oil was decanted. The fat absorption capacity was determined from the difference in weight and expressed as grams of oil absorbed per gram of starch. Fat absorption was calculated from the following equation:

(1) FAC=(10−b)/W

where FAC—fat absorption [%], 10—the number of cm3 of oil used; b—the volume of decanted oil [cm^3^]; and W—the mass of the sample [g].

**Figure 10:**
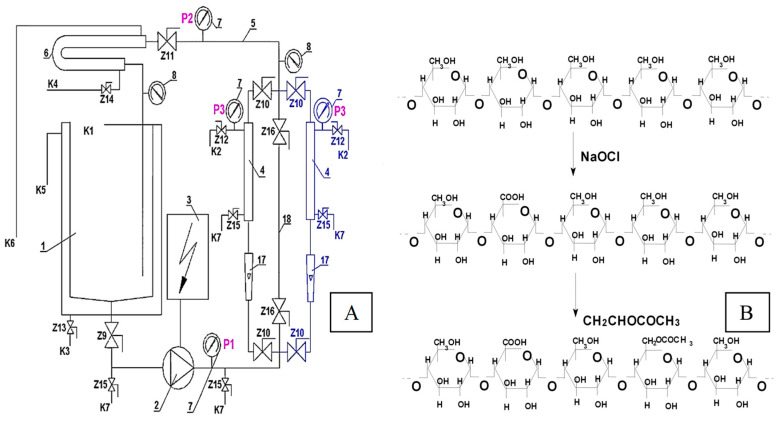
(**A**) Recirculated membrane reactor: 1—reactor tank, 2—circulation pump, 3—power inverter, 4—UF module, 5—pipelines, 6—cooler, 7—manometer, 8—thermometer, 17—rotameter, 18—bypass, K1 ÷ K7—stubs, Z9 ÷ Z16—valves; (**B**) reaction of obtaining acetylated oxidized starch.

The hydrolysis process for doubly modified starch preparations by oxidation and acetylation was carried out in a recirculated membrane reactor (CRMR) (Figure 10A). The reactor consisted of a 5 dm^3^ capacity reaction vessel made of acid- and alkali-resistant stainless steel with a water jacket, an external ultrafiltration module equipped with two ceramic membranes, a bypass for periodic hydrolysis without the simultaneous separation of the reaction mixture, and a flow pump. TAMI ceramic membranes were used in the study, featuring a 3-channel tubular configuration, 250 mm length, and a total surface area of 0.083 m^2^. Membranes with cut-off separation capacities of 8, 15, and 50 kDa were employed.

The hydrolysis process was conducted at a constant temperature of 60 °C, a pH of approximately 6.5, and a transmembrane pressure (Δp) of 0.5 MPa. The initial starch solutions were prepared by heating a starch suspension at 90 °C with continuous stirring (300 rpm). The concentration of the initial modified starch paste depended on the type of starch preparation undergoing hydrolysis. For starch with a degree of substitution (DS) with carboxyl groups of 0.2%, the initial paste concentration was 5%. For preparations with an oxidation degree of 2.5%, the starch paste concentration was 10%. The prepared paste, after cooling to 60 °C, was introduced into the CRMR reactor vessel, and then the enzymatic preparation BAN 480 was added in the amount of 0.3 mL/kg dry matter of starch.

As mentioned, the study utilized a CRMR reactor with a bypass for periodic enzymatic hydrolysis without simultaneous product separation in the UF module (see Figure 10A). In the initial 30 min of hydrolysis, the reaction mixture circulated through the system via the bypass, bypassing the filtration unit. Subsequently, for 150 min, hydrolysis was conducted with the simultaneous two-stage separation of hydrolysates in the UF modules. Two ultrafiltration modules with two sets of membranes were used: the first set employed membranes with cut-offs of 50 and 15 kDa, and the second set used membranes with separation capacities of 15 and 8 kDa. Filtration was initially carried out for 60 min using the membrane with the larger cut-off in the respective set, which was followed by 90 min of separation using the membrane with the smaller cut-off.

The efficiency of the enzymatic hydrolysis process of modified starch conducted in the CRMR was evaluated by measuring the filtrate flux. The effectiveness of hydrolysis was determined by the degree of saccharification of the hydrolysates. During the process, the filtrate flux was measured every 10 min, and every 30 min, a sample of the filtrate fraction was taken to determine the dry matter (DM) content and the average degree of saccharification of the components in the given fraction. The determination of DM was conducted in accordance with the established standard [47]. The content of reducing groups (DE) was determined using the Schoorl–Roenbogen method, following standard [48].

The process of obtaining oxidized and acetylated starches was conducted in two stages (Figure 10B). In the first stage, native starch was subjected to an oxidation reaction, which was carried out at a temperature of 32 °C and a pH of 9.5–10.0 in the presence of sodium hypochlorite as the oxidizing agent. 

In the second stage, the previously oxidized waxy-corn or potato starch was subjected to an esterification process in a batch reactor with acetic anhydride as the acetylating agent. The acetylation process took place at room temperature, and the pH of the suspension, within the range of 8.5–9.5, was adjusted using 3% NaOH.

For all derivatives, analyses were conducted to confirm the degree of substitution with acetyl groups. Additionally, the structure of the starch granules was examined, and the rheological properties of the obtained starch derivatives were studied.

Enzymatic hydrolysis was performed on 3 types of potato starch (designated as P) and 4 types of waxy-corn starch (designated as C). All analyzed starch preparations were chemically modified through oxidation and acetylation, differing in the percentage content of carboxyl groups (0.2% or 2.5%, denoted as 0.2Ox and 2.5Ox, respectively) and acetyl groups (0.5% or 2.5%, denoted as 0.5Ac and 2.5Ac) within the starch macromolecule. The enzymatic hydrolysis process of the doubly modified starch preparations was conducted using BAN 480L enzyme (1,4-alpha-D-glucan glucanohydrolase), in a recirculating membrane reactor equipped with an external ultrafiltration module (Figure 10A).

For the hydrolysis of potato starch, a 5% initial slurry concentration was used for derivatives with low oxidation levels and a 10% concentration for the P-2.5Ox-2.5Ac derivative (due to differences in viscosity of starch pastes). The concentration of waxy-corn starch paste during hydrolysis was consistent at 5%.

The efficiency of hydrolysis was quantified by measuring the change in dry matter content in the filtrate fraction. The degree of saccharification (DE, dextrose equivalent) of the hydrolyzed derivative was employed to measure the effectiveness of the process.

## 4. Conclusions

Comprehensive studies were conducted on the physicochemical and functional properties of chemically modified native potato starch and waxy-corn starch (through oxidation and acetylation) and their enzymatic hydrolysis products, which were processed in a recirculating membrane bioreactor. Contrary to literature reports, all starch preparations resulting from double chemical modification showed no significant changes in starch granule morphology or tendency to aggregate (clump) regardless of the botanical source of the starch. 

Rheological tests on aqueous suspensions/solutions of starch preparations showed that with an increase in the degree of substitution of the starch macromolecule with carboxyl groups, there was a significant decrease in the viscosity of the solutions. The modified starch preparation with a high carboxyl group content exhibited dynamic viscosity that was three orders of magnitude lower than that of starches with a low degree of oxidation. Furthermore, it was demonstrated that only the degree of oxidation of the starch preparation significantly influences paste viscosity, while the introduction of acetyl groups into the starch macromolecule causes a slight change in the solution’s rheology for both potato and waxy-corn starch.

It was demonstrated that the modification process did not alter the flow curve characteristics for derivatives with a low carboxyl group content compared to the original native waxy-corn starch. However, for preparations with a higher carboxyl group content, the modification resulted in a change in the flow curves’ characteristics. The starch pastes were observed to undergo a transition from shear-thinning fluids to shear-thickening fluids. 

It was found that the content of both the acetyl and carboxyl groups in the starch molecule affects the efficiency of the enzymatic hydrolysis process. Hydrolysates obtained from the enzymatic hydrolysis of derivatives with higher acetyl group content showed a lower degree of saccharification than hydrolysates of derivatives with a lower acetyl group content. The cut-off membranes used in the recirculating membrane reactor also significantly influenced the degree of saccharification.

The introduction of functional groups into the starch molecule resulted in a direct increase in its lipophilicity compared to native starch, consequently enhancing its oil absorption capacity. Furthermore, the oil absorption capacity of the hydrolysate was influenced by the duration of the enzymatic hydrolysis process conducted in the CRMR. The carboxyl group content was found to be a determining factor in the water-binding capacity of doubly modified starches. The water retention capacity was found to be greater than that of unoxidized starch preparations with lower carboxyl group content (i.e., derivatives with a low degree of oxidation). 

The swelling power of the examined derivatives was primarily determined by the degree of oxidation. It was observed that the swelling power of starch granules increased dramatically with temperature. It was observed that neither the doubly modified starch preparations nor their hydrolysates exhibited foaming properties regardless of the biopolymer’s botanical origin.

## Figures and Tables

**Table 1:** The content of acetyl groups in modified derivatives.

	Waxy Corn (C)		Potato (P)	
Preparate	%Ac	DS	%Ac	DS
0.2Ox-0.5Ac	0.48	0.02	0.6	0.02
0.2Ox-2.5Ac	2.51	0.1	2.61	0.1
2.5Ox-0.5Ac	0.46	0.02	0.6	0.02
2.5Ox-2.5Ac	2.54	0.1	2.64	0.1

**Table 2:** Viscosity of starch paste.

Parameter	Origin	0.2Ox-0.5Ac	0.2Ox-2.5Ac	2.5Ox-0.5Ac	2.5Ox-2.5Ac
Viscosity η, Pa·s	Waxy corn	0.41	0.33	0.02	0.02
Viscosity η, Pa·s	Potato	0.20	0.21	0.003	0.003

**Table 3:** Change in the viscosity of the retentate during the P-0.2Ox-0.5A starch hydrolysis process provided in CRMR (500 rpm).

Hydrolysis time, min	0	30	60	90	120	150	180	210
Viscosity η, Pa·s	0.184	0.0065	0.0038	0.0036	0.0037	0.0040	0.0038	0.0038

## Data Availability

The raw data supporting the conclusions of this article will be made available by the authors on request.
